# Factors affecting birth preparedness and complication readiness in Jimma Zone, Southwest Ethiopia: a multilevel analysis

**DOI:** 10.11604/pamj.2014.19.272.4244

**Published:** 2014-11-12

**Authors:** Gurmesa Tura Debelew, Mesganaw Fantahun Afework, Alemayehu Worku Yalew

**Affiliations:** 1 Department of Reproductive Health and Health Service Management, School of Public Health, Addis Ababa University, Addis Ababa, Ethiopia; 2 Department of Epidemiology and Biostatistics, School of Public Health, Addis Ababa University, Addis Ababa, Ethiopia

**Keywords:** Birth preparedness, complication readiness, multilevel analysis, Southwest Ethiopia

## Abstract

**Introduction:**

Birth preparedness and complication readiness have been considered as comprehensive strategy aimed at promoting the timely utilization of skilled maternal health care. However, its status and affecting factors have not been well studied at different levels in the study area. Thus, this study was aimed to fill this gap by conducting community based study.

**Methods:**

A cross-sectional study was conducted among randomly selected 3612 pregnant women from June-September 2012. The data were collected by interviewer-administered structured questionnaire and analyzed by SPSS V.20.0 and STATA 13. Mixed-effects multilevel logistic regression model was used to identify factors affecting birth preparedness and complication readiness.

**Results:**

The status of birth preparedness and complication readiness was 23.3% (95% CI: 21.8%, 24.9%). Being in urban residence and having health center within two hours distance were among the higher level factors increasing birth preparedness and complication readiness. Educational status of primary or above, husband's occupation of employed or merchant, third or above wealth quintiles, knowledge of key danger signs during labor, attitude and frequency of antenatal care visits were among the lower level factors found to increase the likelihood of preparation for birth and its complications.

**Conclusion:**

The status of birth preparedness and complication readiness was low in the study area. Both community level and individual level factors had important program implications. Socio demographic, economic, knowledge of key danger signs, attitude and antenatal care use were identified as associated factors. Improving antenatal care, giving special emphasis to danger signs and community based health education are recommended.

## Introduction

Globally, about 287,000 mothers die each year because of problems related to pregnancy and child birth. About 99% of this occurs in developing countries. Sub-Saharan Africa (56%) and Southern Asia (29%) account for 85% of the global burden of maternal death. With this disparity, the maternal mortality rate in developing countries is more than 15 times higher than in the developed regions [1]. While almost all developed countries have achieved the desired three-fourth reduction of maternal mortality by 2015, many of the developing countries are on track. However, majority of the resource limited countries in Sub-Saharan Africa have shown the slowest progress with an average annual rate of decline of 2.6% [2, 3].

Ethiopia is one of the Sub-Saharan African countries with high maternal mortality ratio (MMR) and very slow progress. The MMR in the Ethiopian Demographic and Health Survey (EDHS) 2011 was 676 per 100,000 live births which had shown non-significant decline as compared to 673 and 871 per 100,000 live births in EDHS 2005 and 2000, respectively. As a result, improving maternal health status so as to achieve the intended target is among the top priority areas of the country [4–6]. The high maternal mortality and slow progress in low and middle income countries, in part, are explained by the low coverage of maternal health care and the three delays to health care seeking behaviour of mothers. These problems are again influenced by demographic, poor socio-economic status and poor quality of services [7–9]. To address these problems, birth preparedness and complication readiness (BP and CR) has been considered as a comprehensive strategy aimed at promoting timely utilization of skilled maternal health care and addresses the three delays to care-seeking for obstetric emergencies. It promotes active preparation and decision-making for births, including pregnancy and postpartum periods, by shared responsibilities of pregnant women, their families, the community and the service providers [10, 11].

The elements of birth preparedness and complication readiness at individual level include: plan for where to give birth, plan for a skilled birth attendant, plan to save money, plan for transportation and identification of compatible blood donors in case of emergency. These enable the woman and her family for timely use of skilled maternal and neonatal cares based on the theory that preparing for childbirth and being ready for complications reduce delays in obtaining these cares [10]. Though we have limited studies in Ethiopia, the existing evidences show that the status of birth preparedness and complication readiness is low. A cross sectional study conducted in Adgrat town in 2006 revealed that only 22% of the respondents were prepared for birth and its complications [12]. Similarly, a cross-sectional study conducted in Aleta Wondo District, South Ethiopia in 2007 found that only 17% of the respondents were prepared for birth and its complications [13].

Thus, it is timely and very crucial to have up-to-date information on the status of birth preparedness and complication readiness and affecting factors at different levels for policy and program implications. However, studies on this issue are very limited in Ethiopia and non-existent in the study area. Even, the existing studies didn't look at the factors at different levels. Therefore, this study was conducted to fill this gap by identifying the factors affecting birth preparedness and complication readiness at the different levels by applying multilevel analysis of community based study in Jimma zone.

## Methods

### Study design and setting

This is a community based cross-sectional study conducted in Jimma Zone from June-September 2012. Jimma Zone is one of the 17 Zones of the Oromia Regional State of Ethiopia. The capital of the Zone, Jimma Town, is located at 346kms Southwest of Addis Ababa. The Zone has a total of 17 rural districts ("*Woredas*") and two town administrations. Based on the 2007 national population census conducted by the Central Statistics Agency (CSA) of Ethiopia, the Zone has a total population of 2.6 million, of whom 88.7% are rural inhabitants [14, 15].

### Population, sample size and sampling technique

The study population for this study was pregnant women. The sample size was determined by using Epi-Info V.3.5.1 by considering two sample comparison of proportions based on the following assumptions. Among all the factors considered, antenatal care (ANC) was found to give the largest sample size. The prevalence of birth preparedness and complication readiness among mothers who attended ANC is estimated to be 22% (p_1_=0.22) and among those who didn't attend ANC is to be 13% (p_2_ = 0.13) [13]; 95% level of confidence and 90% power were considered. The prevalence of ANC in the general population in same study was 45%. As a result, a ratio of 1:1 was used (r = 1). As multistage clustered sampling method was used, a design effect of 2 was considered. Finally, 10% was added for non-responses and the final sample size became 1650. However, this study was part (baseline) of a big longitudinal study to determine the effect of maternal and neonatal health care on neonatal health status, in which 3612 pregnant women have been followed up. As a result, all the 3612 pregnant women were included in the analysis for this study.

Multi-stage clustered sampling technique was used to identify pregnant women for the study. At first stage, the Zone was stratified as rural districts (17 in number) and town administrations (2 in number, Jimma and Agaro). Then, 5 districts were selected by simple random sampling from the 17 districts. At second stage, all the selected 5 districts were clustered by “*Kebeles*” (A “*kebele*” is the smallest administrative unit having 5000 population on average) and stratified in to urban and rural “*Kebeles*”. Then, by simple random sampling method, 9 rural “*Kebeles*” and 2 urban “*Kebeles*” were selected from each selected district. This number of “*kebeles*” was determined based on expected number of pregnant women per “*Kebele*. Jimma town administration and Agaro town administration have 13 and 5 “*Kebeles*”, respectively and all were included. With this, a total of 73 “*Kebeles*” (clusters) were included in the study. Then, for all selected “*kebeles*” pregnant women were enumerated by using house-to-house visit and all obtained were included in the study.

### Data collection process

Pre-tested interviewer administered structured questionnaire was adapted from the safe motherhood questionnaire developed by maternal and neonatal health program of JHPIEGO [11]. The indicators for the wealth index were adapted from EDHS [6]. The questionnaire was prepared in English, then translated to local languages “*Afan Oromoo*” and Amharic and back translated to English by different experts to check its consistency. Females, who had completed 10^th^ grade or above, were recruited, trained and collected the data. The data collection process was supervised strictly by trained supervisors and principal investigators. To control the quality of data, in addition to training, pretest, supervision and use of local languages, the inter-item consistency of the indicators to measure the composite score of BP and CR was checked by using Chronbach-alpha at 0.7 cut-off point.

### Data analysis

The collected data were coded and entered into Epidata V.3.1. and exported to SPSS for windows version 20.0 for cleaning, editing and analysis. Descriptive analysis was done by computing proportions and summary statistics. Wealth quintiles were determined by using Principal Component Analysis (PCA). As Jimma town and Agaro town administrations were both purposefully included, the status of BP and CR was estimated by calculating weighted percentage based on the complex sample survey procedure by considering probability of exclusion at each stage and non responses in order to avoid over estimation. Bivariate analysis was done by using cross tabulation to see associations between the independent and dependent variables. Then, all variables having P < 0.25 were considered as candidates for the mixed-effects multilevel logistic regression model. Because of the multi-stage clustered sampling procedure, mixed effects multilevel logistic regression model was used by using STATA 13. This model was preferred in order to avoid the clustering effects as well as ecological fallacy. *“Kebeles”* was considered as clusters and *"kebele"* level variables were taken as higher level (level-2).

Mothers were nested within their households and maternal individual variables and household characteristics were taken as lower level (level-1) variables (Table 1, Table 2). Goodness-of-fit of the multilevel model was tested by the log likelihood ratio (LR) test. To evaluate the existence of sufficient variance at the cluster level to use multilevel analysis, intercept-only model was fitted as Logit (p_ij_) = y_00_+u_0j_ and Interclass Correlation Coefficient (ICC) was determined by dividing between group variance (δ^2^µ_0_) by the total variance variance (between group variance (δ^2^µ_0_)+within group variance (δ^2^e)). However, within group variance (δ^2^e) is not directly obtained for dichotomous outcome variable; instead, it was estimated by Π^2/3^.

**Table 1:** Description of the dependent, level-2 and level-1 predictors, Jimma Zone, Southwest Ethiopia, June-September, 2012 (Part I)

Variables	Descriptions	Measurements
**Dependent variable**		
Birth preparedness and complication readiness	A package of interventions composed of composite measure of 5 variables (planed to save money, planed to arrange transport, identified place of delivery, identified skilled attendant and identified blood donor)	Women were labeled as ‘Yes =1’ if planed otherwise ‘No = 0’. Composite variable was computed by adding the five responses. Women who scored 3 or more ‘Yes’ responses were categorized as ‘prepared’ otherwise ‘not prepared’
**Level-2 predictor variables**	**Communal (kebele) characteristics**	
Place of residence	Women were asked whether they live in urban or rural residents	Urban kebele was coded as ‘1’ and rural kebele was coded as ‘0’.
Average distance from health centre	Women were asked about the approximate distance from the nearest health centre on foot in munities. When they had difficulties, the data collector assisted in estimating the approximate time it takes	Average distance was computed for each kebele and dichotomized as ‘ ≤ 2hours’ and ‘ > 2hours’
Average distance from Hospital	Women were asked about the approximate distance from the nearest hospital on foot in munities. When they had difficulties, the data collector assisted in estimating the approximate time it takes	Average distance was computed for each kebele and categorized as ‘ ≤ 2 hours’, ‘ > 2-12hrours’ or ‘ > 12hours’
**Level-1 predictor variables**	**Individual and household characteristics**	
Age	Age of women at interview was asked in completed years	Categorized in to7 groups by five-years interval, which later recoded in to three categories: ‘ < 20’, ‘20-29’ or ‘ > 29’
Ethnicity	The ethnic background of the women were asked and recorded	Each ethnicity was entered and later recoded as ‘Oromo’ and ‘Others’. Others were merged because they were very few for logistic regressions.
Religion	The religious background of the women were asked	Each religion was entered and later recoded as ‘Muslim’ or ‘Others’. Others were merged because they were very few for logistic regressions.
Educational status	Highest level of education attained by the respondent and her husband	Categorized into 4 groups as ‘no Formal Education’, ‘primary (1-8)’, ‘Secondary (9-12)’ and ‘tertiary (12^+^)’.
Occupational status	Current employment status and specific occupation of respondent and her husband	Categorized as ‘housewife’ (‘farmer’ for husbands), ‘employed’, ‘merchant’ and ‘others’.
Wealth quintiles	Using EDHS questionnaire, household assets ownership were assessed and wealth index was computed by using principal component analysis (PCA)	The wealth status was categorized into five groups and ranked from poorest to wealthiest quintile.

**Table 2:** Description of obstetric related level-2 predictors, Jimma Zone, Southwest Ethiopia, June-September, 2012 (part II)

Knowledge of key danger signs during pregnancy	Spontaneous response to the three key danger signs: severe vaginal bleeding, swollen hands/face and blurred vision were asked	For each key danger sign, ‘Yes’ was coded as ‘1’ and ‘No’ was coded as ‘0’. After adding the three responses, women who responded ‘Yes’ to all the three danger signs (scored 3/3) were labeled as ‘knowledgeable’ and otherwise ‘not knowledgeable’.
Knowledge of key danger signs during labor	Spontaneous response to the four severe vaginal bleeding, convulsions, prolonged labour and retained placenta were asked	For each key danger sign, ‘Yes’ was coded as ‘1’ and ‘No’ was coded as ‘0’. After adding the four responses, women who responded ‘Yes’ to all the four danger signs (scored 4/4) were labeled as ‘knowledgeable’ and otherwise ‘not knowledgeable’.
Knowledge of key danger signs during postnatal period	Spontaneous response to the three key danger signs: severe vaginal bleeding, foul smelling vaginal discharge and high fever were asked	For each key danger sign, ‘Yes’ was coded as ‘1’ and ‘No’ was coded as ‘0’. After adding the three responses, women who responded ‘yes’ to all the three danger signs (scored 3/3) were labeled as ‘knowledgeable’ and otherwise ‘not knowledgeable’.
Knowledge of key danger signs during postnatal period	Spontaneous response to the four key danger signs: difficult or fast breathing, small at birth, lethargy/unconscious and seizure/convulsion were asked	For each key danger sign, ‘Yes’ was coded as ‘1’ and ‘No’ was coded as ‘0’. After adding the four responses, women who responded ‘Yes’ to all the four danger signs (scored 4/4) were labeled as ‘knowledgeable’ and otherwise ‘not knowledgeable’.
Attitude towards BP and CR	Attitude of the respondents was measured by using a composite variable of 8 items in a Likert-scale	The responses were categorized as: ‘1 = strongly disagree’, ‘2 = disagree’, ‘3 = indifferent’, ‘4 = agree’ and ‘5 = strongly agree’. Then, after creating composite variable after adding the 8 questions, mean score was determined. Those who scored above or equal to mean were labeled as having “favorable attitude’ and otherwise ‘non-favorable attitude’.
Gravida	Number of pregnancies a woman ever have including current pregnancy	Categorized in to three: ‘primi gravida’, ‘gravid 2-4’ and ‘gravida 5 or above’
ANC frequency	Having health facility visit for pregnancy check up by skilled attendants during pregnancy.	Categorized in to three: ‘no ANC visit at all’, ‘1-3 ANC visits’ and ‘ ≥ 4 ANC visits’

Then, to identify the determinate factors, the full model was fitted as: Logit (p_ij_)= p_00_+y_01_Z_j_+y_10_X_ij_+u_0j_+u_1j_X_ij_. Where: Logit(p _ij_) = dependent variable at unit i in cluster j, X_ij_= individual explanatory variable in cluster j, Z_j_= group level explanatory variable, y _00_= fixed intercept, y_01_ and y_10_ = fixed slopes and u _0j_ and u_1j_= random effects at level-2. Multicollinearity between the independent variables was assessed by using variance inflation factors (VIF > 10 was considered as suggestive of multicollinearity) before interpreting the final output. But, no significant multicollinearity was detected as VIF for all variables were < 5. In addition cross-level two-way interactions were checked, particularly between place of residence, access to health facilities and ANC visits. However, no significant interaction was detected (P > 0.05 for each).

### Ethical clearance

Ethical approval was obtained from the Institutional Review Board (IRB) of College of Health Sciences of Addis Ababa University. Formal permission letters were secured from all respective local administrators. Written informed consent was obtained from each respondent before actual data collection and confidentiality of the data were strictly maintained.

## Results

### Socio-demographic characteristics

Of the total 3612 pregnant women included, 2716 (75.2%) were rural residents. Majority, 2323 (64.3%), lied in the age group of 20-29, with a mean (±SD) age of 26.5±5.0 years. Oromoo were the predominant ethnic group, 3161 (87.7%). The leading religion was Muslim, 3149 (87.1%). Nearly all, 3589 (99.4%) were in a marital union and more than half, 1955 (54.1%) didn't attend any formal education. The great majority, 3413 (94.5%), were housewives and farmer was the leading occupation of their partners 2566 (71.0%) (Table 3).

**Table 3:** Socio-demographic characteristics of respondents, Jimma Zone, Southwest Ethiopia, June-September, 2012 (n = 3612)

Variables	No.	%
**Residence**		
Urban	896	24.8
Rural	2716	75.2
**Age (Years)**		
15-19	179	5.0
20-24	1023	28.3
25-29	1300	36.0
30-34	766	21.2
35-39	311	8.6
40-44	33	0.9
**Ethnicity**		
Oromo	3161	87.7
Amhara	175	4.8
Dawuro	102	2.8
Others*	174	4.7
**Religion**		
Musilim	3149	87.1
Orthodox	360	10.0
Protestant	103	2.9
**Marital Status**		
In marital union	3589	99.4
Not in marital union†	23	0.6
**Educational status**		
No formal education	1955	54.1
Grades 1-4	843	23.3
Grades 5-8	479	13.3
Grades 9-12	265	7.3
Tertiary (12+)	70	1.9
**Occupation**		
Housewife	3413	94.5
Employed (GO, NGO and Private)	88	2.4
Others‡	111	3.1
**Husband's Occupation**		
Farmer	2566	71.0
Employed (GO, NGO and Private)	398	11.1
Merchant	421	11.7
Others‡	227	6.2

* Yem, Kaficho, Guraghe and Tigrie

† Single, divorced and widowed

‡ Merchant, student, daily laborer

### Past obstetric history

Gravida, number of pregnancies ever had, ranged from 1 to 9 among the respondents with a mean (±SD) of 3.3±1.9. Similarly, number of live births ever had ranged from 1-8 with a mean (±SD) of 2.8±1.6. Among those who ever had two or more pregnancies, 156 (5.5%) and 203 (7.2%) had life time history of abortion and stillbirth, respectively. Nearly four-fifth, 2236 (79.1%) had the last inter-pregnancy interval of 2-4 years with a mean of 3±1.3 years. Among those who had at least one delivery in the past, 1690 (59.8%) had at least one ANC visit and only 406 (14.5%) gave birth attended by skilled providers.

### Knowledge of key danger signs

The three key danger signs during pregnancy: severe vaginal bleeding, swollen hands/face and blurred vision were spontaneously mentioned by 964 (26.7%), 530 (14.7%) and 1078 (29.8%), respectively. However, only 227 (6.3%) were able to spontaneously mention all the three key danger signs. The four key danger signs during labor and delivery: severe vaginal bleeding, convulsions, prolonged labor and retained placenta, were spontaneously mentioned by 1788 (49.5%), 588 (16.3%), 537 (14.9%) and 545 (15.1%), respectively. Very few, 133 (3.7%), were able to mention all the four key danger signs spontaneously.

The three key danger signs during postnatal period: severe vaginal bleeding, foul smelling vaginal discharge and high fever were spontaneously mentioned by 1638 (45.3%), 548 (15.2%) and 436 (12.1%), respectively. However, only 88 (2.4%) were able to mention all the three key danger signs spontaneously. The four key danger signs of neonates: difficult or fast breathing, small at birth, lethargy/unconscious and seizure/convulsion were spontaneously mentioned by 1483 (41.1%), 675 (18.7%), 377 (10.4%) and 243 (6.7%), respectively. While, 75 (2.1%) were able to mention the four key danger signs.

### Attitude towards BP and CR

Attitude of the respondents was measured by using a composite variable of 8 items in a Likert-scale and those achieved above or equal to the mean score were rated as having favorable attitude. Based on this, 2202 (61.0%) were found to have favorable attitude towards birth preparedness and complication readiness.

### BP and CR practice

Among the five key elements of BP and CR practice, 2656 (73.5%) planned to save money, 2174 (60.2%) planned to arrange transport, 719 (19.9%) planned to prepare blood donor, 1169 (32.4%) planned to give birth in health facility and 791 (21.9%) planned to be attended by skilled attendant for their current pregnancy. By considering 3 or more steps of the five parameters, 1245 (34.5%) were found to fulfill the criteria and rated as well prepared for birth and its complications. However, after weighted analysis to avoid over estimation, the final status of BP and CR was found to be 23.3% (95% CI: 21.8%, 24.9%) (Table 4).

**Table 4:** Knowledge of key danger signs, BP & CR practice, Jimma Zone, Southwest Ethiopia, June-September, 2012

Variables	Number (n = 3612)	Unweighted%	Weighted%
**Knowledge of key danger signs**			
know all the three key danger signs during pregnancy	227	6.3	6.4
know all the four key danger signs during labor and childbirth	132	3.7	3.6
know all the three key danger signs during postpartum period	88	2.4	2.0
know all the four key danger signs of neonates	75	2.1	2.0
**Attitude towards BP and CR (composite)**			
Favorable	2202	61.0	62.2
Non favorable	1410	39.0	37.8
**Components of BP and CR practice**			
Planed to save money	2656	73.5	69.1
Planed to arrange transport system	2174	60.2	56.1
Planed to give birth in Health facility	1169	32.4	17.9
Planed to be attended by skilled attendant	791	21.9	14.5
Planed to arrange blood donor	719	19.9	17.5
**Number of steps taken**			
< 3 (Poor preparation)	2367	65.5	76.7
≥ 3 (Good preparation)	1245	34.5	23.3

***Factors affecting birth preparedness and complication readiness*** Before running the full model, ICC (ρ) was calculated in the empty model and it was found to be 0.554 indicating that 55.4% of the variation is contributed by between cluster variation. The test of the preference of log likelihood versus logistic regression was also strongly significant (P < 0.0001). Then, the full model was run by including both the cluster level and individual level variables and the ICC (ρ) was reduced to 0.302. This again indicated that 30.2% of the variation is attributed to cluster level variables suggesting the preference of multilevel analysis. The preference of log likelihood versus logistic regression was again strongly significant (P < 0.0001).

After adjusting for confounders in the two-levels mixed-effects multilevel model, among the cluster level variables, place of residence and access to health center were found to have statistically significant association with BP and CR practice. Women from urban residence (OR = 6.01; 95%CI: 2.56, 14.08) and women who were from clusters (Kebeles) found within 2 hours travel on foot from health centre on the average (OR = 2.93; 95%CI: 1.43, 6.02) were more likely to be prepared for birth and its complications. Among the socio-demographic and economic characteristics considered as level-1, educational status, husband's occupation and wealth quintiles were found to have statistically significant association with BP and CR practice. Women who attended primary (OR = 1.55; 95%CI: 1.24, 1.94), secondary (OR = 3.13; 95%CI: 2.00, 4.91) or tertiary (OR = 8.04; 95%CI: 2.14, 30.24) were more likely to be prepared as compared to women who didn't attend any formal education.

Women having employed (OR= 1.77; 95%CI: 1.14, 2.74) or merchant husbands (OR = 2.04; 95%CI: 1.40, 2.96) were more likely to be prepared as compared to women having farmer husband. Women in the third (OR = 1.46; 95%CI: 1.06, 2.00), fourth (OR = 1.24; 95%CI: 1.06, 1.72) or fifth (OR = 1.56; 95%CI: 1.12, 2.19) wealth quintiles were more likely to be prepared as compared to women in the lowest quintiles (poorest) (Table 5). Among the obstetric related factors considered at individual level, knowledge of key danger signs, attitude and frequency of ANC visits had significant association with BP and CR practice. Women who knew all the four key danger signs during labor and delivery were more likely to be prepared for birth and its complications (OR = 2.04; 95%CI: 1.22, 3.39). Similarly, having favorable attitude towards BP and CR was found to increase the likelihood of preparation significantly (OR = 1.73; 95%CI: 1.37, 2.18). ANC visit was also among the strong predictors of BP and CR. Having 1-3 visits (OR = 2.12; 95%CI: 1.67, 2.69) and greater or equal to 4 visits (OR = 2.87; 95%CI: 1.98, 4.18) were found to increase the likelihood of preparation as compared to those who didn't attend ANC visit at all (Table 6).

**Table 5:** Multilevel analysis of factors affecting BP & CR, Jimma Zone, Southwest Ethiopia, June-September, 2012 ( part I)

Factors	BP and CR Practice			Crude OR (95%CI)	Adjusted OR(95%CI)
	Prepared (n = 1245) n(%)	Not prepared (n = 2367) n(%)	Total (n = 3612) n(%)		
**Higher level variables**					
**Place of residence**					
Rural	580(21.4)	2136(78.6)	2716(100.0)	1.00	1.00
Urban	665(74.2)	231(25.8)	896(100.0)	10.60 (8.90, 12.64)	6.01(2.56, 14.08)
**Average distance from health centre (on foot)**					
≤2 hours	1108(42.9)	1472(57.1)	2580(100.0)	4.92(4.04, 5.98)	2.93(1.43, 6.02)
>2 hours	137(13.3)	895(86.7)	1032(100.0)	1.00	1.00
**Average distance from Hospital (on foot)**					
≤2 hours	359(71.4)	144(28.6)	503(100.0)	6.25(5.08, 7.71)	0.95(0.37, 2.44)
>2 hours	886(28.5)	2223(71.5)	3109(100.0)	1.00	1.00
**Level-1 variables**					
**Socio-demographic and economic characteristics**					
**Age (in years)**					
<20	76(42.5)	103(57.5)	179(100.0)	1.00	1.00
20-29	848(36.5)	1475(63.5)	2323(100.0)	0.78(0.57, 1.06)	1.02(0.64, 1.62)
≥30	321(28.9)	789(71.1)	1110(100.0)	0.55(0.40, 0.76)	1.96(0.69, 1.96)
**Ethnicity**					
Oromo	956(30.2)	2205(69.8)	3161(100.0)	1.00	1.00
Others	289(64.1)	162(35.9)	451(100.0)	4.12(3.35, 5.06)	0.99(0.66, 1.50)
**Religion**					
Muslim	933(29.6)	2216(70.4)	3149(100.0)	1.00	1.00
Others	312(67.4)	151(32.6)	463(100.0)	4.91(3.98, 6.05)	1.25(0.82, 1.91)
**Educational status**					
No Formal Education	438(22.4)	1517(77.6)	1955(100.0)	1.00	1.00
Primary (1-8)	541(40.9)	781(59.1)	1322(100.0)	2.40(2.06, 2.80)	1.55(1.24, 1.94)
Secondary (9-12)	201(75.8)	64(24.2)	265(100.0)	10.88(8.05, 14.69)	3.13(2.00, 4.91)
Tertiary (12+)	65(92.9)	5(7.1)	70(100.0)	45.03(18.02, 112.51)	8.04(2.14, 30.24)
**Occupation**					
House wife	1097(32.1)	2316(67.9)	3413(100.0)	1.00	1.00
Employed	74(84.1)	14(15.9)	88(100.0)	11.16(10.28, 19.85)	0.79(0.32, 2.08)
Others¥	74(66.7)	37(33.3)	111(100.0)	4.22(2.83, 6.31)	0.81(0.45, 1.46)
**Occupation of Husband**					
Farmer	538(21.0)	2028(79.0)	2566(100.0)	1.00	1.00
Employed	306(76.9)	92(23.1)	398(100.0)	12.54(9.75, 16.13)	1.77(1.14, 2.74)
Merchant	281(66.7)	140(33.3)	421(100.0)	7.57(6.05, 9.47)	2.04(1.40, 2.96)
Others¥	120(52.9)	107(47.1)	227(100.0)	4.23(3.20, 5.58)	1.15(0.71, 1.85)
**Wealth Index**					
1st Quintile (lowest)	157(21.7)	565(78.3)	722(100.0)	1.00	1.00
2nd Quintile	249(34.4)	474(65.6)	723(100.0)	1.89(1.50, 2.41)	1.23(0.90, 1.68)
3rd Quintile	250(3.6)	472(65.4)	722(100.0)	1.91(1.51, 2.41)	1.46(1.06, 2.00)
4th Quintile	255(35.3)	468(64.7)	723(100.0)	1.96(1.55, 2.48)	1.24(1.06, 1.72)
5th Quintile (highest)	334(46.3)	388(53.7)	722(100.0)	3.10(2.46, 3.90)	1.56(1.12, 2.19)

¥ Daily laborer or student

**Table 6:** Multilevel analysis of obstetric factors affecting BP & CR, Jimma Zone, Southwest Ethiopia, June-September, 2012 (part II)

Level-1: obstetric related variables	BP and CR Practice			Crude OR (95%CI)	Adjusted OR(95%CI)
	Prepared (n = 1245) n (%)	Not prepared (n = 2367) n(%)	Total (n = 3612) n(%)		
**Knowledge of key danger signs during pregnancy**					
Not knowledgeable	1139(33.6)	2246(66.4)	3385(100.0)	1.00	1.00
Knowledgeable	106(46.7)	121(53.3)	227(100.0)	1.73 (1.32, 2.26)	1.24(0.83, 1.84)
**Knowledge of key danger signs during labor**					
Not knowledgeable	1169(33.6)	2311(66.4)	3480(100.0)	1.00	1.00
Knowledgeable	76(57.6)	56(42.4)	132(100.0)	2.68(1.89, 3.82)	2.04(1.22, 3.39)
**Knowledge of key danger signs during post natal period**					
Not knowledgeable	1198(34.0)	2326(66.0)	3524(100.0)	1.00	1.00
Knowledgeable	47(53.4)	64(46.6)	88(100.0)	2.23(1.46, 3.40)	1.67(0.89, 3.12)
**Knowledge of key danger signs of neonates**					
Not knowledgeable	1200(33.9)	2337(66.1)	3537(100.0)	1.00	1.00
Knowledgeable	45(60.0)	30(40.0)	75(100.0)	2.91(1.83, 4.66)	1.10(0.60, 2.04)
**Attitude toward BP and CR**					
Unfavorable attitude	337(23.9)	1073(76.1)	1410(100.0)	1.00	1.00
Favorable attitude	908(41.2)	1294(58.8)	2202(100.0)	2.23(1.93, 2.60)	1.73(1.37, 2.18)
**Gravida**					
Primi	375(47.8)	410(52.2)	785(100.0)	1.00	1.00
2-4	664(35.0)	1234(65.0)	1898(100.0)	0.58(0.50, 0.70)	0.88(0.67, 1.16)
>4	206(22.2)	723(77.8)	929(100.0)	0.31(0.25, 0.38)	0.72(0.51, 1.03)
**ANC Visit**					
Not at all	182(15.4)	996(84.6)	1178(100.0)	1.00	1.00
1-3 times	870(41.4)	1229(58.6)	2099(100.0)	3.87(3.24, 4.64)	2.12(1.67, 2.69)
≥4 times	193(57.6)	142(42.4)	335(100.0)	7.44(5.69, 9.73)	2.87(1.98, 4.18)

In the empty model: ICC = 0.554 = 55.4%, LR Vs χ^2^ test (P < 0.0001)

In the full model = 0.302 = 30.2%, LR Vs χ^2^ test (P < 0.0001)

## Discussion

As the occurrences of complications during the process of child birth are unpredictable, every woman needs to be aware of the key danger signs of obstetric complications during pregnancy, delivery and the postpartum period. This knowledge will ultimately empower them and their families to make prompt decisions to seek care from skilled birth attendants [11]. However, the knowledge of key danger signs in this study was found to be very low. The three key danger signs during pregnancy, the four key danger signs during labor and delivery, the three key danger signs during postpartum period and the four key danger signs of neonates were spontaneously mentioned by 6.3%, 3.7%, 2.4% and 2.1%,respectively which were very low. Similar problems were reported in other prior studies in Ethiopia and other African countries [12, 16–18].

This low level of knowledge of key obstetric danger signs in developing countries may be explained by the low coverage of ANC visits and inadequate number of visits. This may also indicate that less attention might have been given to key danger signs while giving health education and advice during ANC. In this study, 54.1% didn't attend any formal education and the other 36.6% attended only primary education. This low level of education might have also limited their access to information and contributed to this low level of knowledge.

In this study, 73.5% of respondents planned to save money and 60.2% planned to arrange transport which were relatively better. However, only 19.9% planned to arrange blood donor, 32.4% planned to give birth in health facility and 21.9% planned to be attended by skilled attendants which were still low. These low levels of preparations were also reported in other prior studies in the country. In the study conducted in Adgrat Town, North Ethiopia in 2006, 35.6% saved money, 3.2% identified a mode of transportation, 39.1% identified place of delivery and 10.5% identified skilled attendant at delivery [12]. Similarly, in the study conducted in Sidama Zone, South Ethiopia in 2007, 34.5% planed to save money, 7.7% planed to arrange transport, 2.3% planed to arrange blood donor and 8.1% planed to deliver in health facilities [13]. This may be explained by the low socio-economic status, low level of knowledge and low education among women as well as the general population. As birth preparedness and complication readiness is relatively a recent strategy, service providers and program planers might not have given special attention.

Particularly, the preparation of health facility delivery and skilled delivery attendant, were lower than some other countries’ study like Tanzania [18], Nigeria [19] and Burkina Faso [20]. This difference may be explained by the difference in socio-economic status, availability and accessibility of services or any other interventions in the countries. The overall status of birth preparedness and complication readiness in this study was 23.3%. This is consistent with other priori studies in the country. In a study done in Adgrat town, North Ethiopia in 2006 and Sidama Zone, South Ethiopia in 2007, the status of birth preparedness and complication readiness were 22% and 17%, respectively [12, 13]. This suggests that in spite of many efforts by the government, the progress is slow. In this study, the adjusted final multilevel model indicated 30.2% of the variation is explained by the cluster level variables. This means that 69.8% of the variation is contributed by the household and individual level factors. This indicated that both the community level and individual level factors were found to be important for program implications. However, more emphasis should be given to the individual and household level factors while addressing the problem of BP and CR.

Among the higher level factors, being in urban residence was found to increase the likelihood of preparation for birth and its complication by about six times in this study. Having health centers within 2 hours distance on foot in average was also found to increase the likelihood of preparation by about three times. These findings are consistent with other previous studies [12, 18]. This may be explained by the fact that women in urban areas have access to health information through different media and relatively low barriers due to distance and lack of transportations as compared to the rural women. Similarly, having nearby health facility increases access to health information and also reduces distance barriers. Among socio-demographic and economic related factors, education, husband's occupation and wealth quintiles were the detrminats of BP and CR in this study. Attending primary (grades 1-8), secondary (grades 9-12) and tertiary (12+) schools were found to increase the likelihood of preparation by about 1.5, 3 and 8 times, respectively. Similarly, having partner who is employed or merchant increased the likelihood of preparation for birth and its complication as compared to those whose partners were farmers. Besides, wealth quintiles were found to be the other determinant factor. Being in the wealth quintile category of third and above were associated with increased preparation for birth and its complications. This is in line with the findings of other study conducted in the country as well as other countries; where education, occupation and income were among the factors affecting birth preparedness and complication readiness [12, 18]. The reasons could be, having high education, being employed and merchant are directly related with high access to information and income which enable them to be prepared for birth and its complications.

Most importantly, Knowledge of key danger signs during labor and delivery, women's attitude towards BP and CR and ANC visit were found to be the determinant factors in this study. However, knowledge of key danger signs during pregnancy, post partum period and neonates were not found to have significant associations. This is in line with other studies in the country and abroad in which ANC visit and knowledge of danger signs were among the strong predictors of BP and CR practice [12, 13, 18]. This might be due to the advice about the risks of pregnancy and importance of BP and CR given during ANC. The health education during ANC also increases knowledge of danger signs and develops favorable attitude that enhance preparation for birth and its complications. This study may have its own limitation because of its cross-sectional nature to ascertain temporal relationship. In addition, some variables like wealth quintiles and indicators of attitude were subjective. Dichotomizing such composite variable may have its own limitations in identifying cut-off points. Possible efforts were made to use standard and as many variables as possible to make them objective.

## Conclusion

This study revealed that the level of knowledge of key obstetric danger signs and the status of birth preparedness and complication readiness in the study area were very low. Place of residence, access to health centre, educational status, husband's occupation, wealth quintiles, knowledge of key danger signs, attitude and ANC visit were identified as factors affecting birth preparedness and complication readiness. Improving ANC, giving special emphasis to knowledge of key danger signs and BP and CR during health education and ANC counseling are recommended as a short term solutions. Women education, job and income generating activities to raise the socio-economic status of the women are recommended as long term interventions.
